# Advances in research on severe fever with thrombocytopenia syndrome virus

**DOI:** 10.3389/fmicb.2025.1622394

**Published:** 2025-07-23

**Authors:** Yu Yu, Jing Li, Qingqing Liu, Rongrong Zhai, Yuzhu Dai, Lei Sun

**Affiliations:** ^1^Department of Clinical Laboratory, Lu'an People's Hospital, Lu'an, China; ^2^Department of Clinical Laboratory, Fuyang Second People's Hospital, Fuyang, China; ^3^Department of Clinical Research, The 903rd Hospital of PLA, Hangzhou, China; ^4^Department of Clinical Laboratory, Qilu Hospital of Shandong University, Jinan, China

**Keywords:** severe fever with thrombocytopenia syndrome virus, genotype, clinical features, pathogenesis, rapid detection

## Abstract

Severe fever with thrombocytopenia syndrome (SFTS) is an acute infectious disease caused by the SFTS virus (SFTSV). Since the first reported case, SFTSV has spread globally, particularly in Asian regions such as China, South Korea, and Japan, with an increasing number of cases and a high mortality rate among severe patients. SFTSV is an RNA virus capable of rapid biological evolution through genetic mutations, reassortment, and homologous recombination. The disease primarily occurs in mountainous, forested, and hilly areas. Due to limited clinical research, the clinical characteristics and pathogenesis of SFTS remain incompletely understood. This review summarizes recent advances in the regional epidemiological characteristics, clinical features, genotyping, pathogenesis, and rapid detection methods of SFTSV.

## 1 Introduction

Severe fever with thrombocytopenia syndrome (SFTS) is an acute infectious disease characterized by fever, thrombocytopenia, and leukopenia (Seo et al., 2021). The virus isolated from the serum of SFTS patients, initially named Huaiyangshan virus, was later reclassified as Dabie bandavirus (DBV) in 2019, belonging to the *Phenuiviridae* family and *Bandavirus* genus (Niu et al., 2024). SFTSV is primarily transmitted through tick bites but can also spread via contact with infected blood or bodily fluids (Kim et al., 2024; Kim and Park, 2023). Although the International Committee on Taxonomy of Viruses (ICTV) has reclassified the virus, the terms “SFTSV” and “SFTS” remain widely used. For consistency with previous studies, this review will use “SFTSV” and “SFTS” to refer to the virus and the disease, respectively.

SFTSV is an enveloped virus with a diameter of 80–120 nm, containing three single-stranded negative-sense RNA segments: large (L), medium (M), and small (S), with lengths of 6,368 bp, 3,378 bp, and 1746 bp (Wang et al., 2022; Liu et al., 2016). The complementary ends of the genome form a circular structure. The L segment encodes the RNA-dependent RNA polymerase (RdRp), the M segment encodes a membrane protein precursor that is cleaved into Gn and Gc proteins, and the S segment is a bicistronic RNA encoding the non-structural protein (NSs) and the nucleocapsid protein (NP) (Williams et al., 2023). The SFTSV Gn and Gc exist as a heterodimer on the surface of viral particles, and further assemble into pentameric and hexameric peplomers with the dimer as the structural unit, thus constituting a virus particle similar to an icosahedron (Du et al., 2023). This indicates that adjacent Gn/Gc dimers form a closely packed structure rather than a simple dimeric form. After SFTSV infection, viral particles bind to receptors on the surface of host cells through their membrane glycoproteins Gn and Gc. Studies have shown that dendritic cell-specific intercellular adhesion molecule-3-grabbing non-integrin (DC-SIGN) and Nonmuscle Myosin Heavy Chain IIA (NMMHCIIA) promote viral adsorption by recognizing the glycosylation sites of Gn (Spiegel et al., 2016; Yuan and Zheng, 2017). After SFTSV binds to receptors on the surface of host cells, it enters the host cells through a clathrin-dependent endocytic pathway (Liu et al., 2019). In a low pH environment, the Gc protein undergoes a conformational change, exposing the fusion loop. The fusion loop of Gc inserts into the endosomal membrane of the host cell, promoting the fusion of the viral envelope with the endosomal membrane (Wu et al., 2017). After membrane fusion is completed, the viral genomic RNA is released into the cytoplasm of the host cell, and NP and RdRp work together to initiate the replication and transcription of the viral genome. The above processes indicate that the glycoproteins Gn and Gc play a major role in viral replication. They are also important targets for specific neutralizing antibodies (Chang et al., 2024). In addition, during the process of viral entry into host cells, Gc mediates the fusion of the viral envelope with the endosomal membrane. In this process, some Gc subunits may dissociate from the dimeric structure and participate in processes such as membrane fusion in an independent form. Under low pH conditions, Gc may exist in the form of independent subunits to form trimers, promoting the fusion of the virus with the host cell membrane (Yuan and Zheng, 2017). NP and RdRp form ribonucleoprotein complexes (RNPs) that protect the virus from degradation by nucleases and the host immune system. NP and NSs play important roles in evading host immune responses and promoting viral replication. NP can inhibit the RIG-I/MDA5 pathway to block IFN production (Min et al., 2020). SFTSV NSs are potent IFN antagonists, which exert inhibitory effects on IFN by binding to several host molecules and sequestering them into inclusion bodies (IBs) (Ren et al., 2021).

Since its first identification, SFTSV has spread widely, particularly in China, South Korea, and Japan, posing a significant public health threat (Li et al., 2021). The transmission dynamics and pathogenesis of SFTSV are not fully understood (Casel et al., 2021). What is certain is that the segmented nature of the SFTSV genome allows for homologous recombination and reassortment between different genotypes during viral replication, which enhances viral genetic diversity and leads to the emergence of new viral strains, thereby facilitating rapid viral spread (Kwon et al., 2024). Furthermore, previous studies have shown that different SFTSV genotypes exhibit significant differences in pathogenicity and case fatality rates. Therefore, unified and accurate genotyping of SFTSV holds important practical significance for the selection of clinical treatment approaches and the implementation of public health interventions (Dai et al., 2022). This review will summarize the regional epidemiological characteristics, clinical features, genotyping, pathogenesis, and rapid detection methods of SFTSV.

## 2 Regional distribution of SFTS

**Figure 1:**
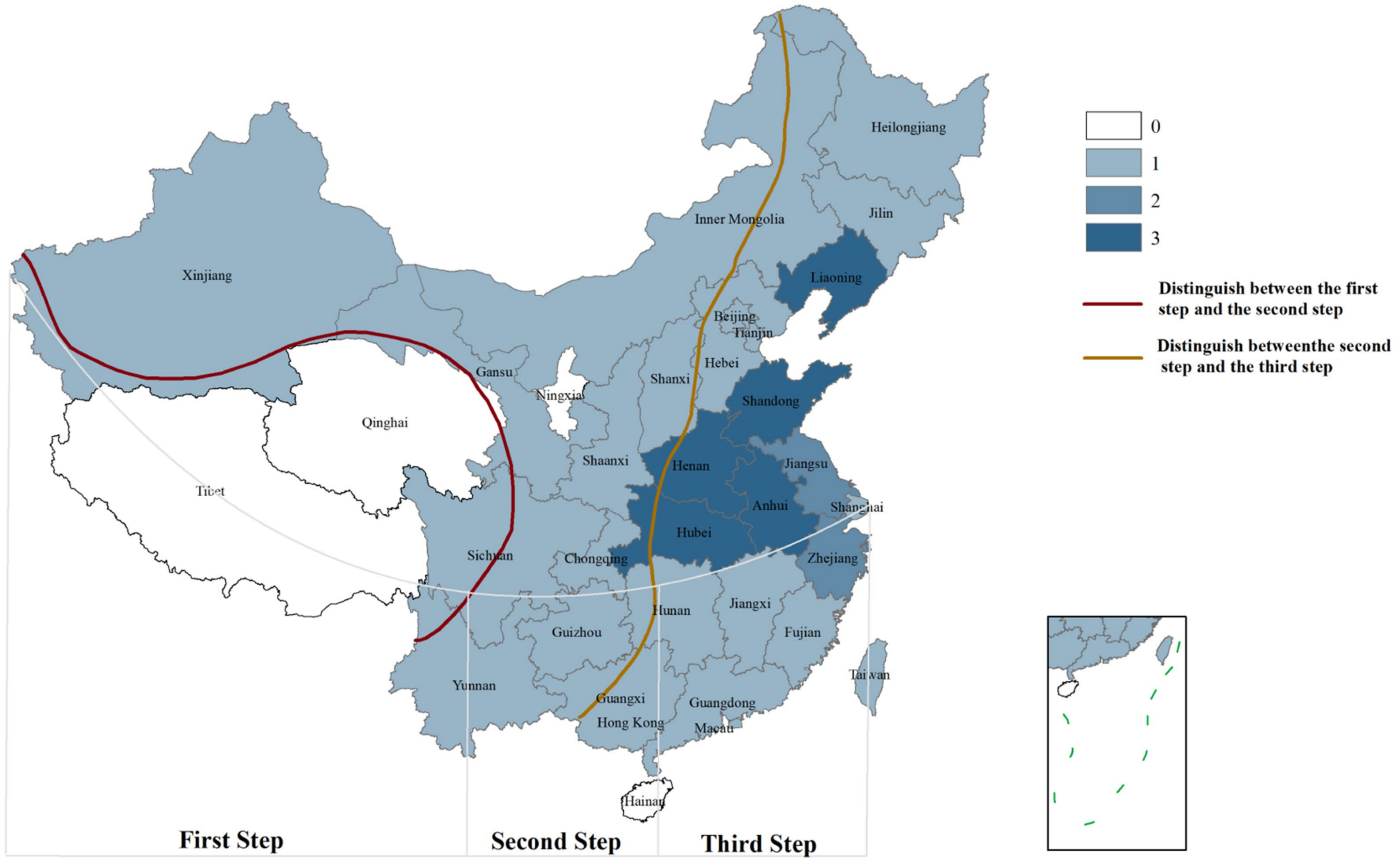
Geographical distribution of SFTS cases in China. 0: Area with 0 cases of infection; 1: Area with 1–100 cases of infection; 2: Area with 101–1,000 cases of infection; 3: Area with>1,000 cases of infection. First Step: Average altitude above 4,000 m; Second Step: Average altitude above 1,000-2000 m; Third Step: Average altitude below 500 m [The data comes from the study of Chen et al. (2022), created by Arcmap V10.2.2].

SFTSV is transmitted through tick bites. *Haemaphysalis longicornis* is widely recognized as the primary vector, followed by *Haemaphysalis flava*, *Rhipicephalus microplus*, *Amblyomma testudinarium*, *Dermacentor nuttalli*, *Hyalomma asiaticum*, and *Ixodes nipponensis* (Casel et al., 2021; Zhu et al., 2019). Ticks have a broad host range, and SFTSV is thought to circulate in a tick-animal-tick transmission cycle. Currently, SFTSV RNA or anti-SFTSV antibodies have been detected in wild animals such as hedgehogs, rodents, and some bird species, as well as domestic animals like cattle, sheep, and pigs (Chen et al., 2019; Huang et al., 2019; Zhao et al., 2022). This indicates a high zoonotic transmission potential of SFTSV. Additionally, studies have shown that exposure to body fluids and secretions of infected patients can lead to SFTSV infection, suggesting human-to-human transmission. SFTSV infections predominantly occur from spring to autumn, with higher incidence rates in people living in mountainous, forested, and hilly areas or working outdoors—consistent with the main habitats of ticks (Peng et al., 2025). In high-altitude areas (averaging over 4,000 m), the spread of SFTSV is restricted due to the reduced geographical distribution of ticks and low population density, which is consistent with the results of SFTS case distribution shown in Figure 1. Tick growth and reproduction are closely associated with climatic factors, including light, humidity, and temperature (Pérez et al., 2024). For example, *H. hystricis* prefers warm and humid environments, while *H. longicornis* exhibits stronger environmental adaptability, widely distributed in rural landscapes and urban areas (Miao et al., 2020). Changes in environmental factors, particularly climatic-ecological and geographical landscape factors, may provide suitable ecological conditions for natural tick population growth, contributing to the seasonal variation characteristics of SFTSV infections. Furthermore, host animals carrying ticks expand their survival range through natural migration, further accelerating cross-regional transmission of SFTSV (Ji et al., 2024).

The reported cases of SFTS are mainly concentrated in East Asia. SFTS was first reported in central China between March and July 2009, with subsequent cases reported across various provinces. Japan reported its first case in 2012, primarily in eastern and southern regions, while South Korea reported its first fatal case in 2013, although infections likely occurred earlier (Liu et al., 2014). Most cases are concentrated in China, South Korea, and Japan, with sporadic reports from other regions. Retrospective analyses of serum samples from patients with acute febrile illnesses in Southeast Asian countries such as Vietnam and Thailand have detected SFTSV (Tran et al., 2019; Rattanakomol et al., 2022). SFTSV has also been detected in *Rhipicephalus microplus* and domestic poultry in Taiwan, China (Lin et al., 2020). Additionally, in the United States, cases infected with Heartland virus recorded in 2009 showed disease progression similar to SFTSV infection. Heartland virus and SFTSV are both in the *Bandavirus* genus within the *Phenuiviridae* family, *Bunyavirale*. Field sampling and laboratory work identified *Amblyomma americanum* as the vector of Heartland virus (Staples et al., 2020; Godsey et al., 2016).

In China, the national reported incidence of SFTS has shown an upward trend. As of 2023, the cumulative number of reported cases and deaths nationwide has reached 27,447 and 1,326 respectively, affecting 27 provinces. The average case-fatality rate is 4.83% (Yue et al., 2024; Chen et al., 2022; Huang et al., 2024). SFTSV cases are predominantly clustered in mountainous, forested, and hilly areas of central China, although reports from other regions are increasing, raising concerns about potential public health crises (Liang et al., 2023; Huang et al., 2021). In Japan, epidemiological surveys from 2013 to 2017 identified 310 cases, with an average mortality rate of 7.8%, and 60 to 100 new cases are added annually (Saijo, 2022; Crump and Tanimoto, 2020; Kobayashi et al., 2020). In South Korea, 1,203 cases and 231 deaths were reported by August 2020 (Jang et al., 2024). The reporting rate of SFTS in South Korea is on the rise, with a fatality rate of 18.54% (Cui et al., 2024). The geographical distribution of SFTS cases in China is illustrated in Figure 1. Additionally, seroprevalence studies have revealed significant regional variations. A meta-analysis of SFTSV antibody prevalence in China reported an overall seroprevalence of 4.3%, indicating widespread transmission and the presence of unreported mild or asymptomatic infections (Li et al., 2017). In contrast, the seroprevalence in Japan is much lower, ranging from 0.14 to 0.3% (Maslow et al., 2019). A seroscreening trial for SFTSV conducted in rural areas of South Korea by Han *et al* showed an SFTSV IgG antibody seroprevalence rate of 4.1%, slightly lower than that in China (Han et al., 2020). These differences may reflect variations in regional transmission patterns, such as the predominance of different genotypes or differences in antibody detection methods.

## 3 Clinical features of SFTS

The initial clinical symptoms of SFTSV infection are persistent high fever and respiratory or gastrointestinal symptoms, followed by a gradual decrease in platelets and white blood cells (Li et al., 2021). These symptoms are similar to those of other infectious fevers caused by different pathogens, lacking specificity, and often lead to inappropriate treatment, resulting in disease progression to severe cases and even death. SFTS cases mainly distribute in the population aged 35–80. In SFTS patients, the incidence increases with age, and most fatal cases occur in patients over 50 years old, suggesting that age is a risk factor related to both incidence and mortality (Casel et al., 2021). Asymptomatic carriers also exist in SFTSV infections. DU *et al* found asymptomatic SFTSV infections in serological surveys of healthy populations, which may have an important impact on the dynamics of SFTS outbreaks (Du et al., 2019). Currently, the research on the transmission capacity of asymptomatic carriers is insufficient. Asymptomatic carriers may provide the possibility for the continuous existence of SFTSV in the population and play a non-negligible role in the SFTSV transmission chain. Future work should focus on studying the potential factors for the development of asymptomatic SFTSV infections and preventing transfusion safety to avoid the spread of SFTS epidemics by asymptomatic cases (Zeng et al., 2015). The typical course of SFTSV infection is generally divided into three stages: the fever stage, the multiple-organ dysfunction (MOD) stage, and the convalescence stage (Yuan and Zheng, 2017). The fever stage can last for 5–11 days, during which patients present with influenza-like symptoms, including persistent high fever (body temperature 38–41°C), myalgia, and gastrointestinal symptoms such as anorexia, vomiting, and diarrhea, accompanied by thrombocytopenia (<100.0 × 10^9^/L), leukopenia (<4.0 × 10^9^/L), and lymphadenopathy. The high viral load during the fever stage is an important means for clinical confirmation. Patients typically progress from fever to MOD within 3–5 days. The progression of MOD is rapid, initially affecting the liver and heart, followed by the lungs and kidneys. Rapid elevations in clinical biochemical markers, including serum alanine aminotransferase (>40 IU/L), aspartate aminotransferase (>40 IU/L), creatine kinase (>200 IU/L), creatine kinase isoenzyme (>25 IU/L), lactate dehydrogenase (>245 IU/L), and activated partial thromboplastin time (>43.5 s), indicate liver and kidney dysfunction, myocardial damage, and coagulation disorders (Zhang et al., 2024). Patients with a gradual decline in serum viral load or self-limiting infections enter the recovery stage, with approximately 85% of patients having a good prognosis and their biochemical indicators returning to normal within 3–4 weeks (Zhang et al., 2024). In contrast, clinical laboratory indices continue to rise, including viral load (>1 × 10^6^ copies/mL), activated partial thromboplastin time (>62.6 s), aspartate aminotransferase (>288 IU/L), etc. Elderly patients with underlying diseases who develop neurological symptoms, disseminated intravascular coagulation (DIC), and multiple organ failure (MOF) are more likely to die (Liang et al., 2024).

Additionally, previous studies have reported atypical and special cases of SFTS infection. Yun et al. (2019) compared the chest radiographs and CT scans of SFTS patients and scrub typhus patients, showing that SFTS patients mainly presented with cardiac enlargement, with or without pericardial effusion and patchy consolidation with ground-glass opacity (GGO), while scrub typhus presented with interstitial pneumonia on chest radiographs, which helps in early differentiation between SFTS and scrub typhus. Although most cases present with leukopenia, occasional leukocytosis has been observed in SFTS patients, possibly due to secondary infections. Lee et al. (2024) reported a case of thrombocytopenia with leukocytosis. PCR and antibody titer tests confirmed SFTS, while blood culture results indicated an *Escherichia coli* infection, suggesting that the patient had SFTS complicated by *E. coli* bacteremia. SFTS complicated by encephalitis may be due to the presence of SFTSV in cerebrospinal fluid, with patients presenting with headache and epilepsy and other central nervous system symptoms. Although these central nervous system symptoms have only been reported in a few cases, they are believed to be related to disease severity and death. However, the mechanism by which SFTSV causes central nervous system symptoms remains to be further investigated.

## 4 Pathogenesis of SFTS

The pathogenesis of SFTS is not yet fully understood. A common pathogenic feature of bunyaviruses is their ability to inhibit the host immune response, facilitating rapid viral replication. As an antiviral cytokine, IFN induces multiple antiviral responses to inhibit viral replication (Schneider et al., 2014). The IFN pathway comprises two stages: IFN induction and signal transduction. IFN induction detects viruses through pattern recognition receptors (PRRs) identifying pathogen-associated molecular patterns (PAMPs), while IFN signal transduction is activated by secreted IFN binding to relevant receptors expressed on adjacent cells, leading to antiviral protein expression (Lee and Ashkar, 2018). SFTSV interferes with IFN-I production via multiple mechanisms. During the IFN induction stage, SFTSV NSs protein specifically traps tripartite motif-containing protein 25 (TRIM25) into inclusion bodies (IBs), hinders TRIM25-mediated Lys-63 ubiquitination and RIG-I activation, and suppresses the production of interferon-stimulated genes (ISGs) (Min et al., 2020; Liu et al., 2019). Studies also show that the C-terminal of SFTSV NS protein specifically binds to TANK-binding kinase 1 (TBK1) to form IBs. These IBs not only serve as viral replication sites but also sequester key proteins in the IFN signaling pathway, such as TBK1, NF-κB kinase inhibitor (IKK), and IFN regulatory factor-3 (IRF-3), thereby blocking IFN-I production and promoting viral replication (Khalil et al., 2021; Chen et al., 2017). Additionally, SFTSV NSs protein captures mitochondrial antiviral signaling protein (MAVS) into IBs, disrupting IFN signal transduction and inhibiting NF-κB signaling pathway activation (Wuerth and Weber, 2016).

During the IFN signal transduction stage, SFTSV NS protein suppresses IFN signaling and ISG expression by sequestering STAT2 into IBs and impairing STAT2 heterodimer phosphorylation and nuclear translocation (Kitagawa et al., 2018). It also inhibits exogenous IFN-*α*-induced Jak/STAT signaling by suppressing STAT1 phosphorylation and activation, thereby blocking type I and III IFN signaling (Chen et al., 2017). Further research indicates that SFTSV NS protein hijacks STAT1 into viral IBs and reduces its expression, inhibiting type II IFN responses (Chen et al., 2023). These evidences indicate that SFTSV NSs is an effective IFN antagonist, which exerts inhibitory effects on IFN by binding to several host molecules and sequestering them into IBs, such as RIG-I, TBK1, IKK, IRF3, TRIM25, STAT1 and STAT2.

**Figure 2:**
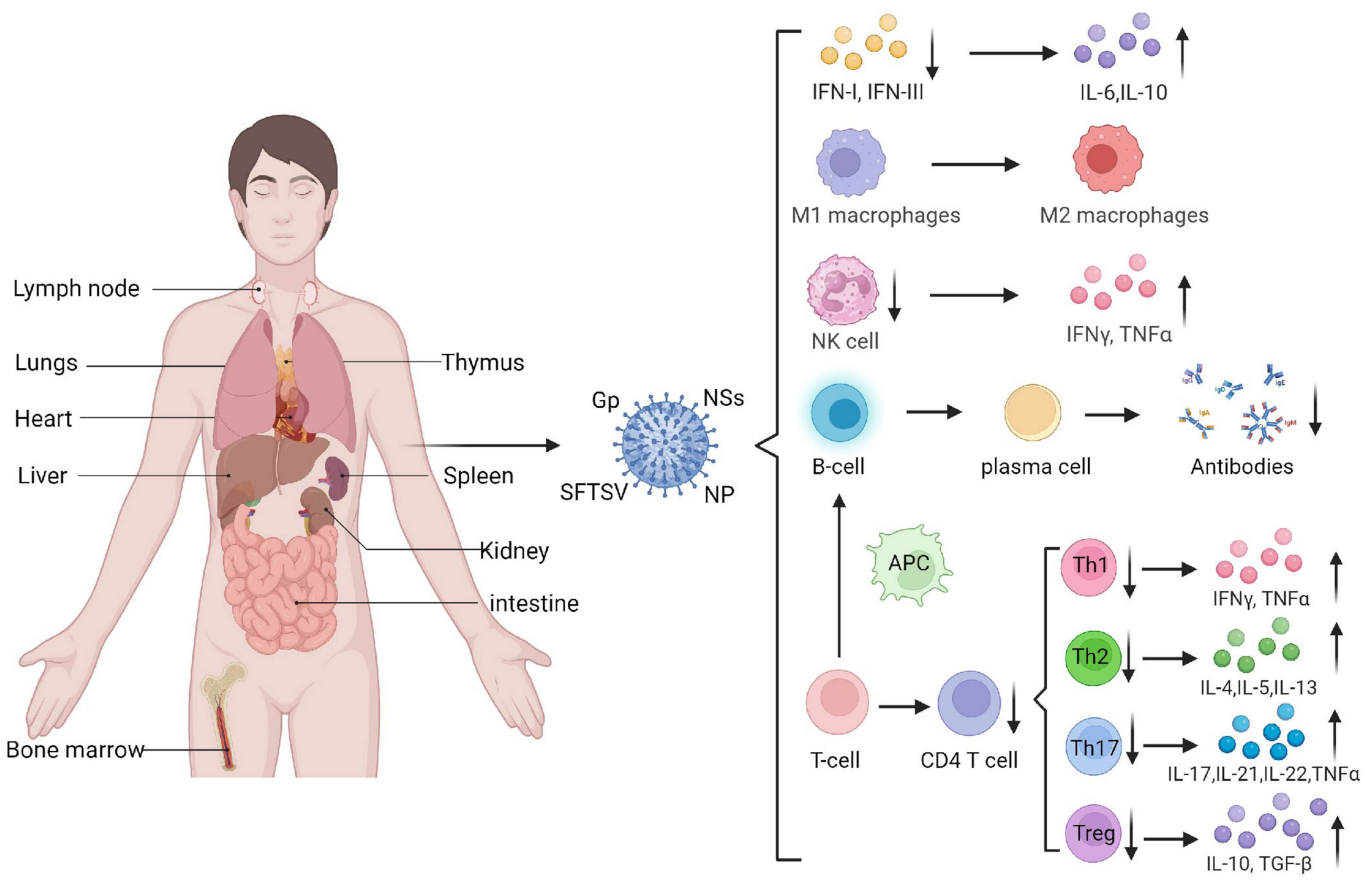
The target organs of SFTSV infection and its impact on immune cells. SFTSV infection impairs the production and function of various immune cells and cytokines, such as IFN, macrophages, and NK cells. It can also suppress the secretion and maturation of T cells and B cells, mediating the occurrence of a cytokine storm (Created by biorender.com).

SFTSV evades immunity by influencing immune responses of immune cells through diverse mechanisms. The main target organs of SFTSV include the spleen, lymph nodes, liver, and bone marrow, while the lungs, kidneys, and heart can also be affected. Macrophages in the spleen and liver are likely the primary target cells for SFTSV infection (Xu et al., 2021) (Figure 2). SFTSV activates STAT1 to induce monocyte immune responses and stimulate macrophage differentiation into the M1 phenotype, leading to inflammatory cytokine production. However, post-infection, it suppresses M1 macrophage differentiation and drives macrophages toward the M2 phenotype, promoting viral shedding and transmission (Zhang et al., 2019). Natural killer (NK) cells control viral load by releasing perforin, granzyme, proinflammatory cytokines, and chemokines to induce host immune responses (Jost and Altfeld, 2013). Studies show that CD3^−^CD16^+^56^+^ NK cells significantly decrease in the early stage of SFTSV infection, indicating their involvement in early immune responses. Depletion of NK cells may contribute to disease progression (Li et al., 2020).

Adaptive immune responses to the virus involve T and B cells, which specifically recognize and eliminate viral pathogens. T lymphocytes are the main cells mediating cellular immunity. Li et al. (2018) found that the number of T lymphocytes, especially CD4^+^ T cells, significantly decreases in SFTS patients, with the reduction magnitude positively correlated with disease severity. Further analysis of naive CD4^+^ T cell subsets showed that the counts of helper T (Th)1, Th2, and regulatory T (Treg) cells in SFTSV fatal cases were significantly lower than those in surviving patients. Increases in Th2 and Th17 cells within CD4^+^ T cell populations lead to abnormal Th1/Th2 and Th17/Treg ratios, which correlate positively with disease severity. Elevated Th17 cells can suppress effector T cell-mediated killing of target cells, hindering host antiviral responses. Additionally, research (Hou et al., 2014) shows that Th17 cells upregulate anti-apoptotic molecules to promote persistent viral replication in SFTSV-infected cells. Thus, SFTSV evades immunity by suppressing T cell responses and regulating infected cell apoptosis.

B cell responses are regulated by antigen-presenting cells and Tfh cells. However, early infection-induced apoptosis reduces dendritic cell (DC)-mediated antigen presentation, impairing Tfh differentiation and function, which significantly weakens humoral immunity. Studies indicate that peripheral blood mononuclear cells in SFTS patients contain transient plasmablasts, and *in vitro* induction shows these atypical lymphocytes are activated B cells, suggesting that SFTSV-infected B cells release factors driving B cell differentiation into plasmablasts (Wada et al., 2022). Most SFTSV in lethal infections resides in plasma B cells, and these activated/differentiated B cells do not express IgM or IgG, leading to insufficient humoral responses in fatal cases. Collectively, these findings show that SFTSV infection suppresses antibody secretion and B cell maturation, thereby evading effective humoral immunity against viral escape from the host immune system (Figure 2).

The cytokine storm induced by SFTSV infection is also believed to play an important role in worsening the condition of SFTS patients. When the host immune response fails to suppress viral replication, SFTSV can induce the release of large amounts of cytokines from target cells, leading to pathological lesions. In the cytokine storm, abnormal expression of several inflammatory cytokines, including IL-6, IL-8, and IL-10, is associated with the severity of SFTS (Song et al., 2024). He et al. (2021) found that monocyte chemoattractant protein-1 (MCP-1), macrophage inflammatory protein-1*α* (MIP-1α), and transforming growth factor-β1 (TGF-β1) were significantly elevated in severe SFTS patients compared to asymptomatic SFTS patients. These cytokines induce abnormal inflammatory disorders, exacerbating multi-organ damage in the body. The low expression of platelet-derived growth factor and secretory factors stored in platelets may be related to thrombocytopenia in peripheral blood, and these cytokines return to normal ranges when patients enter the recovery stage (Li et al., 2021). The bleeding tendency in SFTS patients is also related to elevated tumor necrosis factor (TNF-α). TNF-α acts on endothelial cells, inducing vasodilatory substances, stimulating nitric oxide synthase, increasing capillary endothelial permeability, affecting coagulation function in patients, and thereby increasing the risk of DIC (Xing et al., 2018). Cytokine secretion can also be regulated by multiple cellular signaling pathways, such as JAK/STAT3, MAPK, NF-κB, mTOR, and TLR4 signaling pathways. The crosstalk between these different signals can modulate cytokine expression. These previous findings indicate that cytokine/chemokine-mediated inflammatory responses, characterized by imbalances in cytokine and chemokine expression, play a crucial role in the progression of SFTS.

## 5 Genotyping of SFTSV

Takahashi et al. (2014) conducted a retrospective analysis of SFTS cases in Japan, showed that all Japanese SFTSV isolates clustered into two separate branches from those of Chinese SFTSV isolates, consistent with geographical distribution, suggesting independent natural transmission of SFTSV in Japan. Shi et al. (2017) further included 139 SFTSV isolates from Japan, South Korea, and China in GenBank for phylogenetic analysis. They clustered into five separate clades designated as C1, C2, C3, C4, and J. The study indicated that SFTSV isolates in China and Japan evolved independently within their respective regions. Lv et al. (2017) further divided SFTSV into genotypes C1-C5 and sublineages J1-J3 based on whole-genome sequence analysis for homologous recombination and gene reassortment. Additionally, two new sublineages, C6 and J4, were identified in the S gene segment of SFTSV.

Following the publication of SFTSV sequences from more regions, SFTSV has demonstrated broader genetic diversity. A six-genotype classification (A-F) is now widely used. Fu et al. (2016) classified SFTSV into six genotypes: A, B, C, D, E, and F. The prevalence of different SFTSV genotypes varies among countries. The main genotypes in China are A, D, E, and F, with genotype E being unique to China. In contrast, genotype B is only found in South Korea, Japan, and other regions. Yun et al. (2020) further divided genotype B SFTSV into three subtypes, B1, B2, and B3, based on genetic distance in South Korea. Park et al. (2024) identified the presence of genotype B4 in China, South Korea, and Japan and emphasizing its potential importance in viral evolution. It is noteworthy that there is a significant correlation between SFTSV genotype and mortality rates in different regions. The case fatality rate (CFR) of genotype B2 is the highest among genotype B and its subtypes, at 47.9%. The CFRs of genotypes D and F in China range from 17.3 to 22.7%, while genotype E has a CFR of only 12.5%, and this trend also exists in recombinant strains (Moon et al., 2023). The distribution of SFTSV genotypes in China, South Korea, and Japan is shown in Table 1.

**Table 1:** SFTSV genotype distribution.

Country	Takahashi et al. (2014)	Lv et al. (2017)	Fu et al. (2016)	Yun et al. (2020)	Park et al. (2024)
China	C1, C2, C3, C4, J, Unknown	C1, C2, C3, C4, C5, C6, J3, Unknown	A, C, D, E, F, Unknown	A, C, D, E, F, Unknown	A, C, B3, B4, D, E, F, Unknown
Korea	C3, J, Unknown	C3, C6, J1, J2, J3, Unknown	B, D, F, Unknown	B1, B2, B3, D, F, Unknown	B1, B2, B3, B4, D, F, Unknown
Japan	J, Unknown	J4, Unknown	B, C, Unknown	B2, C, Unknown	B2, C, Unknown

“Unknown” means there is a genetic recombination event and the genotype has not been identified.

Pérez et al. (2024) classified SFTSV genomic evolution into three lineages. Lineage I comprised predominantly Chinese sequences (~95%), while Lineages II and III consisted mainly of South Korean variants. Clade II additionally contained Japanese isolates (~39%), with Lineage III incorporating Chinese and Thai sequences (~32%). Extensive homologous recombination was shown to accelerate SFTSV genomic evolution. The R624W and R962S mutations in the SFTSV glycoprotein precursor (GP) mediate pH-dependent cell membrane fusion (Tsuda et al., 2017; Tani et al., 2019). The N1891K mutation in the RNA-dependent RNA polymerase (RdRp) enhances enzymatic activity (Noda et al., 2020). The NS protein mutations P102A and K211R impair TPL2 signalling and IL-10 production, consequently reducing viral pathogenicity. These findings demonstrate that extensive genomic variations in SFTSV may contribute to regional differences in the pathogenesis and mortality rates of Severe Fever with Thrombocytopenia Syndrome (SFTS) (Ma et al., 2025).

## 6 Rapid detection of SFTSV

Quantitative real-time reverse transcription polymerase chain reaction (qRT-PCR) can be used to detect viral load in patients’ blood and serves as a predictive indicator for disease. However, since the increase in viral load during the early stage of SFTS infection occurs later than the onset of clinical symptoms, timely diagnosis is often not possible in clinical practice, leading to disease progression. Therefore, early and accurate diagnosis is crucial for effective treatment and disease management. Jang et al. (2022) developed a point-of-care molecular diagnostic method based on loop-mediated isothermal amplification (LAMP) technology to differentiate SFTSV infection from scrub typhus, with results available within 30 min and high specificity and sensitivity. Recombinase polymerase amplification (RPA) is an isothermal nucleic acid amplification technique that can rapidly and sensitively detect viral nucleic acids under constant temperature conditions. Zhou et al. (2020) evaluated the performance of RT-RPA for detecting SFTSV in serum samples from suspected SFTS patients, with sensitivity and specificity of 96.00 and 98.95%, respectively, showing good application prospects. Serological detection involves detecting SFTSV-specific antibodies in patients’ serum or plasma to diagnose the disease. Huang et al. (2019) developed an up-converting phosphor technology-based lateral flow (UPT-LF) detection method, coating SFTSV recombinant N protein on a biosensor to detect total SFTSV antibodies in serum through fluorescence signal collection, which can be used for on-site rapid detection. Zuo et al. (2018) developed a lateral flow immunochromatographic strip for ultra-sensitive detection of SFTSV nucleocapsid protein NP, with a detection limit as low as 1 ng/mL, suitable for rapid detection in remote areas.

RT-PCR is the most widely used molecular method for SFTSV diagnosis, offering high specificity and sensitivity. However, its primary limitations include the requirement for expensive thermal cycling equipment and specialised laboratory personnel, restricting its application in resource-limited settings (Zeng et al., 2024). Isothermal amplification technologies, such as LAMP and RPA, eliminate the need for costly thermal cyclers by enabling rapid amplification at a single fixed temperature. When coupled with simple readout devices, these methods facilitate rapid diagnosis in low-resource environments, such as primary healthcare facilities or field screenings in endemic areas. Nevertheless, isothermal amplification generates substantial by-products, particularly non-specific amplification, resulting in relatively lower sensitivity compared to other detection methods (Rolando et al., 2024). Serological assays for detecting SFTSV-specific antibodies are suitable for large-scale epidemiological screening and rapid field testing in resource-limited areas due to their speed, low cost, and minimal equipment requirements (Varghese et al., 2023). However, cross-reactivity with related viruses may lead to false-positive results, while false negatives can occur during the early infection window (Piantadosi and Kanjilal, 2020; Chan et al., 2022). Thus, serological testing should be supplemented with molecular methods to improve diagnostic accuracy. Although significant progress has been made in detecting SFTSV, there is still much work to be done in standardization, automation, and the development of multiplex detection methods to improve detection efficiency and accuracy (Ai et al., 2023).

## 7 Conclusion

SFTSV infection has become a global public health issue. Early diagnosis of SFTS based on typical clinical features and laboratory findings is crucial for improving patient survival rates in clinical practice. Further research on the pathogenesis of SFTS will help elucidate the mechanisms of DIC and MOF to reduce mortality and develop new therapeutic molecules. Comparative studies of viral isolates from different regions may clarify the genetic diversity and variation characteristics of SFTSV. Additionally, the development of rapid detection methods for SFTSV will aid in rapid diagnosis to contain and prevent viral spread.
